# Development of a Method to Detect Three Monohydroxylated Polycyclic Aromatic Hydrocarbons in Human Urine by Liquid Chromatographic Tandem Mass Spectrometry

**DOI:** 10.1155/2015/514320

**Published:** 2015-04-20

**Authors:** Xiaotao Zhang, Hongwei Hou, Wei Xiong, Qingyuan Hu

**Affiliations:** China National Tobacco Quality Supervision & Test Center, No. 2 Fengyang Street, Zhengzhou, Henan 450001, China

## Abstract

A liquid chromatographic tandem mass spectrometry method (LC-MS/MS) for the simultaneous determination of 1-hydroxypyrene (1-OHP), 3-hydroxybenzo[a]pyrene (3-OHBaP), and 3-hydroxybenz[a]anthracene (3-OHBaA) in human urine has been developed. With the exception of 3-OHBaP at a low spiking level, the average recoveries were greater than 80%. The method has good accuracy (72.1–107.7%) and reproducibility (1.8–11.4%) and was successfully used to study the uptake of pyrene, benzo[a]pyrene, and benzo[a]anthracene from cigarette smoke. The results indicated that urinary 1-OHP concentration in the smoking group (66.58 ± 70.91 ng/g creatinine) was higher than that observed in the nonsmoking group (58.16 ± 49.48 ng/g creatinine). Urinary 3-OHBaA concentrations in nonsmokers and smokers with 8 mg and 10 mg tar cigarettes were 10.98 ± 4.39 ng/g creatinine, 11.01 ± 13.30 ng/g creatinine, and 9.17 ± 12.89 ng/g creatinine, respectively. Urinary 3-OHBaP concentrations in nonsmokers and smokers with 8 mg and 13 mg tar cigarettes were 1.30 ± 0.20 ng/g creatinine, 2.83 ± 1.78 ng/g creatinine, and 6.00 ± 4.44 ng/g creatinine, respectively. Urinary 1-OHP levels exhibited a significant correlation with BaP yield in cigarette smoke under the Canadian intense smoking condition (*y* = 3.5563*x* + 30.171, *R* ^2^ = 0.9916, *n* = 227).

## 1. Introduction

Polycyclic aromatic hydrocarbons (PAHs) mainly from incomplete combustion of organic matter are an important class of environmental contaminants, which is found in food [1–3], feed [4, 5], drinking water [6], and so forth. PAHs are also present in cigarette smoke. Pyrene, benzo[a]pyrene, and benz[a]anthracene are thought to be the most common PAHs in cigarette smoke. Benzo(a)pyrene, benz(a)anthracene, and pyrene have been classified as category 1, category 2B, and category 3 carcinogens, respectively, by IARC [7]. In addition, they have been identified as priority environment pollutants by the U.S. Environmental Protection Agency. PAHs in the environment field have been the focus of attention; there are a number of related researches to be reported. For example, Professor Simal-Gandara established the methods to measure PAHs in various foods by the optimization of pretreatment method, such as solid-phase extraction [6] or ultrasound-assisted solvent extraction [8], investigated their potential sources [5, 9], and studied the removal method of PAHs in fish oils [2] and organic solvents [10]. People may be exposed to pyrene, benzo[a]pyrene, and benz[a]anthracene through inhalation, ingestion, or skin absorption. In vivo, pyrene, benzo[a]pyrene, and benz[a]anthracene are metabolised to 1-hydroxypyrene (1-OHP), 3-hydroxybenzo[a]pyrene (3-OHBaP), and 3-hydroxybenz[a]anthracene (3-OHBaA), respectively, which are excreted in urine. Those three urinary monohydroxylated PAHs (OH-PAHs) are commonly used as biomarkers to monitor human PAHs exposure and assess the environmental and human health risks [11, 12].

High performance liquid chromatography (HPLC) with fluorescence detection has been widely used in the determination of urinary OH-PAHs, particularly 1-OHP [13–17]. This method has many advantages including that the instruments are available in most laboratories, the fluorescence detection is highly sensitive, and good specificity for PAH metabolites can be achieved. However, these methods often require fairly large urine specimens (10 mL or more) and long HPLC run times, and most do not employ an internal standard, the use of which is generally expected to result in better precision and accuracy [18–20].

Gas chromatograph (GC) and gas chromatography-mass spectrometer (GC-MS) methods have also been used to detect OHPAHs [21–23]. Recently, high resolution GC-MS methods have been used for the simultaneous determination of several PAH metabolites, demonstrating high sensitivity and specificity [24, 25]. However, time-consuming sample pretreatment and derivatization steps are required prior to GC-MS analysis.

Owing to the fact that LC tandem mass spectrometry (LC-MS/MS) can be used to quantify a wide range of substances in complex biological matrixes at low levels with high specificity and short chromatographic run times, LC time-of-flight MS with electrospray ionization (ESI) [26] and LC-MS/MS with ESI [27] have been used to detect 1-OHP, and a LC-MS method utilizing a single quadrupole instrument with ESI has been used to determine phenolic PAH metabolites in vitro [28]. Recently, certain OHPAH metabolites have been selectively quantified in human urine using LC-MS/MS [29–32]. However, those methods focused only on 1-OHP or 3-OHBaP. To date, simultaneous measurements of urinary 1-OHP, 3-OHBaP, and 3-OHBaA using LC-MS/MS are sparse.

In this study, we describe a LC-MS/MS method for simultaneous determination of urinary 1-OHP, 3-OHBaP, and 3-OHBaA. The proposed method has good precision and accuracy, very high sensitivity, an extraction procedure suitable for processing large batches of samples, and a relatively short chromatographic run time (12 min). This method was successfully applied to determine 1-OHP, 3-OHBaP, and 3-OHBaA levels in urine samples obtained from 81 smokers and 58 nonsmokers.

## 2. Materials and Methods

### 2.1. Chemicals and Reagents

1-OHP was obtained from Dr. Ehrenstorfer GmbH (Augsburg, Germany, purity 98.5%). 3-OHBaP was purchased from the Institute for Reference Materials and Measurements (Belgium, European Commission, purity 99.4%). 3-OHBaA was purchased from MRIGlobal (Kansas City, Missouri, purity >97%) and the D_9_-1-OHP internal standard (chemical purity 97%, isotopic purity 99%) was purchased from Toronto Research Chemicals Inc. (Toronto, Canada). *β*-Glucuronidase-arylsulfatase (30 U/mL *β*-glucuronidase, 60 U/mL sulfatase) was purchased from Merck (Darmstadt, Germany). HPLC-grade acetonitrile was obtained from TEDIA Company Inc. (Ohio, USA).

### 2.2. Standard Solutions of PAH Metabolites

Approximately 2 *μ*g/mL of 1-OHP and 3-OHBaP was dissolved/diluted in acetonitrile to obtain separate stock solutions of each compound. 3-OHBaA was diluted in the same way to a concentration of approximately 1 *μ*g/mL. The D_9_-1-OHP (internal standard) solution was diluted in acetonitrile to concentrations of 50 *μ*g/mL as a stock solution and 100 ng/mL as a working solution. Aliquots of the three standard stock solutions were mixed, and 100 *μ*L of internal standard solution (100 ng/mL) was added. This solution was further diluted with acetonitrile to obtain a series of standard solutions ranging within 0.50–25.00 ng/mL for 1-OHP, 0.10–5.00 ng/mL for 3-OHBaP, and 0.25–12.50 ng/mL for 3-OHBaA. All stock and standard solutions were stored at −80°C.

### 2.3. Assay Development and Validation

The method was validated for specificity, matrix effects, precision, accuracy, linearity, sensitivity, recovery, and stability according to US Food and Drug Administration (FDA) guidelines for the validation of bioanalytical methods [33]. The specificity of this method was determined using three individual human blank urine samples. The matrix effects on the ionization efficiency of each analyte were evaluated by comparing the peak response of the analyte dissolved in pooled blank sample extract (i.e., the final solution obtained from the pooled blank urine sample after extraction and reconstitution) with that of the analyte diluted to the same concentration in the mobile phase (as a reference). The calibration curves were constructed via linear regression using a weighing factor of 1/*C*, where *C* is the concentration of each calibration standard. Correlation coefficients (*r* values) were required to be greater than or equal to 0.99. The limits of quantification (LOQs) were calculated using the background noise level estimated from the peak-to-peak baseline near the analyte peaks. The precision and accuracy of the method were assessed by intra- and interday validation experiments. The intra- and interday accuracy and precision were obtained by determining the concentrations of 1-OHP, 3-OHBaP, and 3-OHBaA in five replicates of pooled blank urine samples for three separate batches. Precision was expressed as the relative standard deviation (RSD). Accuracy was expressed in terms of bias as the percent deviation of the mean determined concentration against the spiked concentration. The recoveries of 1-OHP, 3-OHBaP, and 3-OHBaA were determined by comparing the analyte responses obtained from pooled blank urine samples with those obtained from prepared urine samples that were spiked with equivalent analyte concentrations. Freeze-thaw stability was assessed by exposing samples with moderate concentrations to three freeze-thaw cycles. In each cycle, the samples were removed from the freezer, thawed (unassisted) to room temperature, kept at room temperature for 2 h, and refrozen at −80°C for 8 h.

### 2.4. Instruments and Conditions

Samples were analyzed by an Agilent 1200 rapid-resolution liquid chromatography instrument (autosampler G1367D, binary gradient pump G1312B, column oven G1316B; Agilent Technologies, Palo Alto, CA) on a Phenomenex Synergi Hydro-RP column (2.0 × 100 mm, 2.5 *μ*m particle size, California, USA) and equilibrated with 40% solvent A (water) and 60% solvent B (acetonitrile) at 380 *μ*L/min. The gradient conditions were 0–2.5 min, 40% A; 2.5–3.5 min, 10% A; 3.5–8 min, 10% A; 8–8.1 min, 90% A; 8.1–11 min, 90% A; 11–11.1 min, 40% A; 11.1–12 min, 40% A.

An API 5500 triple quadruple mass spectrometer (Applied Biosystems, Foster City, CA) was used in APCI positive ionization mode. Mass detection conditions were as follows: ion spray voltage, 5500 V; ion source temperature, 400°C; nebulizer gas, nitrogen, 60 psi; curtain gas, nitrogen, 25 psi; collision gas (CID), nitrogen, setting 8. The MRM transitions of* m/z* 219.2→189.1,* m/z* 269.1→252.1,* m/z* 245.1→215.1, and* m/z* 228.2→198.1 were optimized for the quantitation of 1-OHP, 3-OHBaP, 3-OHBaA, and IS, respectively;* m/z* 219.2→201.1,* m/z* 269.1→239.1, and* m/z* 245.1→226.2 were used as confirmation ions for 1-OHP, 3-OHBaP, and 3-OHBaA, respectively.

### 2.5. Urine Sample Collection and Preparation

81 Chinese smokers and 58 Chinese nonsmokers from the city of Zhengzhou were enrolled in the study. For each subject, data regarding smoking habits, age, and job description were collected using a questionnaire administered by trained interviewers. The clinical portion of this study was conducted by the Institute of Clinical Pharmacology of Zhengzhou University in 2010. The study was approved by the Institutional Review Board for Zhengzhou University, China. All participants in the study gave written consent. The ages and occupations of the nonsmokers matched those of the smokers (Table 1). The study was conducted during three separate periods, each including two consecutive days. Every smoker smoked an appointed brand of Chinese Virginia cigarettes and switched to a cigarette of a different tar yield in the next experimental period. All 81 smokers smoked three brands of cigarettes with different tar yields (8, 10, and 13 mg/cigarette) over the three nonconsecutive periods. Urine samples were collected from each smoker every 24 h on three nonconsecutive days and stored at −80°C until analysis.

Urine (10 mL) from each subject was transferred to a conical flask (25 mL). The pH of the solution was adjusted to 5.0 with 0.2 M HCl, and 2.5 mL of 0.5 M acetate buffer (pH 5.0) and 100 *μ*L of internal standard solution (100 ng/mL) were added. After the addition of 30 *μ*L *β*-glucuronidase/arylsulfatase, the conical flask was covered with aluminum foil and placed in a shaker bath overnight at 37°C to completely hydrolyze the conjugated PAH metabolites.

The optimization of the experimental parameters associated with the retention and elution of PAH metabolites (from the solid-phase cartridge) resulted in the following procedures. Aqueous metabolite solutions were processed through a Supelco-ENVI C-18 cartridge (500 mg, 3 mL) that was previously conditioned with 5 mL methanol and 5 mL water. All solutions were injected onto the cartridge with the aid of a 10 mL syringe (attached to the cartridge). The cartridge was sequentially rinsed with 10 mL water and 10 mL of 30% methanol in water. After the cartridge was dried completely, the trapped metabolites were eluted with 4 mL of methanol. The elution was evaporated to dryness under nitrogen gas and redissolved in 150 *μ*L of methanol. The solution was filtered through a 0.22 *μ*m filter and then stored at −20°C until LC-MS/MS analysis.

### 2.6. Determination of Urinary Cotinine and Creatinine

Urinary cotinine levels were analyzed according to a previously published LC-MS/MS method [34]. Briefly, 250 *μ*L urine samples were pipetted into separate polypropylene tubes, and 25 *μ*L of the internal standard solution (10 *μ*g/mL) and 725 *μ*L of water were added. The mixed samples were vortex-mixed for 2 min. After centrifugation for 15 min, the supernatants were filtered through a 0.22 *μ*m syringe filter. 5 *μ*L of the filtrates was then introduced into the LC-MS/MS system.

Urinary creatinine levels were analyzed according to a previously published LC-MS/MS method [35]. Briefly, 10 *μ*L of formic acid was added to a 1 mL aliquot of human urine sample, stirred, and centrifuged at 10000 rpm for 10 min. The mixture was filtered through a 0.22 *μ*m polyethersulfone membrane. 5 *μ*L of urine aliquot was transferred to an amber volumetric flask and brought to a total volume of 10 mL with water after being spiked with 100 *μ*L of creatinine-d_3_ internal standard solution (1 *μ*g/mL). 5 *μ*L aliquot was then injected on-column for LC-MS/MS analysis.

### 2.7. Determination of BaP Yields in Mainstream Cigarette Smoke

BaP yields under different machine smoking regimes using the appointed brands of cigarettes with different tar yields (8, 10, and 13 mg/cigarette) were analyzed according to the reported method [36]. Cigarette smoking was carried out with a SM450 linear smoking machine (Cerulean, UK) according to ISO 3308:2000 [37] and the Canadian intense machine smoking regime. The Canadian intense smoking regime was as follows. Each cigarette was smoked at 1 puff/30 s; each puff had a duration of 2 s and a volume of 55 mL with 100% filter ventilation blocking. Typically, five cigarettes were smoked per port for the ISO regime, and three cigarettes were smoked per port for the Canadian intense regime. All samples were smoked to a butt mark of 3 mm past the tipping paper overwrap.

## 3. Results

### 3.1. Method Validation

Calibration standard solutions were prepared by diluting standard solutions to the appropriate concentrations with pooled blank human urine while a 100 *μ*L internal standard solution was added. The calibration standard solutions were also processed with SPE using the same procedure applied to the real urine samples. The concentrations of the calibration standard solutions (in urine) were 0.008, 0.015, 0.030, 0.076, 0.152, and 0.379 ng/mL for 1-OHP; 0.002, 0.003, 0.006, 0.015, 0.030, and 0.076 ng/mL for 3-OHBaP; and 0.004, 0.008, 0.015, 0.038, 0.076, and 0.19 ng/mL for 3-OHBaA. All urine samples were stored at −80°C until analysis. Each analyte exhibited good linearity with a coefficient of determination (*r*) exceeding 0.99. Data analysis resulted in the following representative regression equations: *Y* = 0.454*X* + 2.06 for 1-OHP, *Y* = 0.215*X* + 0.236 for 3-OHBaP, and *Y* = 1.23*X* − 0.013 for 3-OHBaA, where *Y* indicates the analyte/IS ratio and *X* indicates the urine concentration. The slopes of these regression equations were reproducible for calibration curves determined on three separate occasions (over 3 days). Under optimized conditions, the LOQs (determined when the RSD for precision and the bias for accuracy were both less than 20%, and the signal/noise ratios were approximately 10) were 0.003 ng/mL for 1-OHP, 0.002 ng/mL for 3-OHBaP, and 0.004 ng/mL for 3-OHBaA.

Table 2 demonstrates that the method was accurate, precise, and reproducible for 1-OHP, 3-OHBaP, and 3-OHBaA detection over the tested concentration ranges. The extraction recoveries and the RSD values determined for the analytes (in urine, *n* = 6) are also shown in Table 2. At low spiking concentration, 3-OHBaP recoveries were lower than 80%, whereas the recoveries of the other analytes were always greater than 80% at low, middle, and high spiking levels. All analytes were found to be stable in urine when stored at −80°C for 30 days. No measurable loss of the analytes was observed after three freeze/thaw cycles.

**Figure 1:**
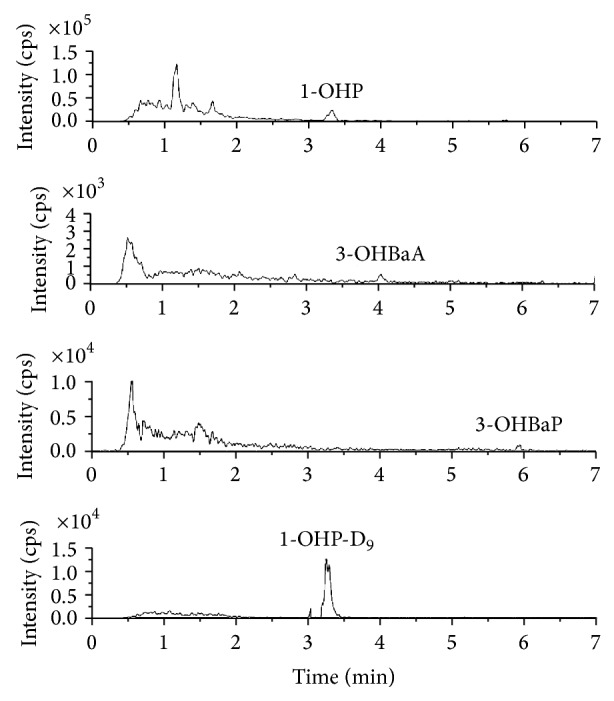
Representative chromatogram from a nonsmoker.

**Figure 2:**
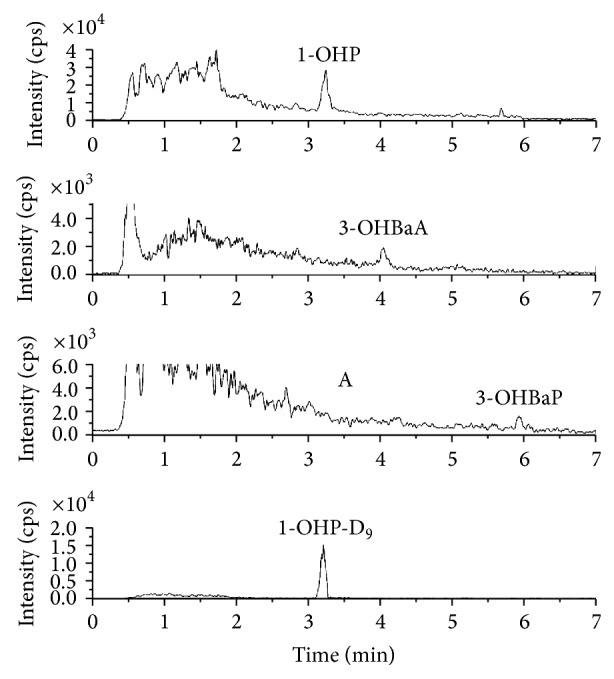
Representative chromatogram from a smoker.

To determine the effects of endogenous interferences, the specificity was assessed by comparing the chromatograms of analyte-free human urine samples with those of the spiked urine samples. Under the described chromatographic conditions, the retention times of 1-OHP, 3-OHBaP, 3-OHBaA, and IS were 3.44, 5.94, 4.31, and 3.29 min, respectively. Representative chromatograms from a nonsmoker and smoker are shown in Figures 1 and 2, respectively. No interfering peaks were observed in the chromatograms of the blank urine samples extracted from the nonsmoker. Matrix interference, caused by endogenous materials in the urine samples, was evaluated by comparing the peak areas of spiked standards with those of the standards of pooled blank urine concentrations. The ratios obtained were within the acceptable limits. No significant ion suppressions or enhancements were observed at the expected retention times of the targeted ions.

### 3.2. Urinary Excretion of 1-OHP, 3-OHBaP, and 3-OHBaA

**Figure 3:**
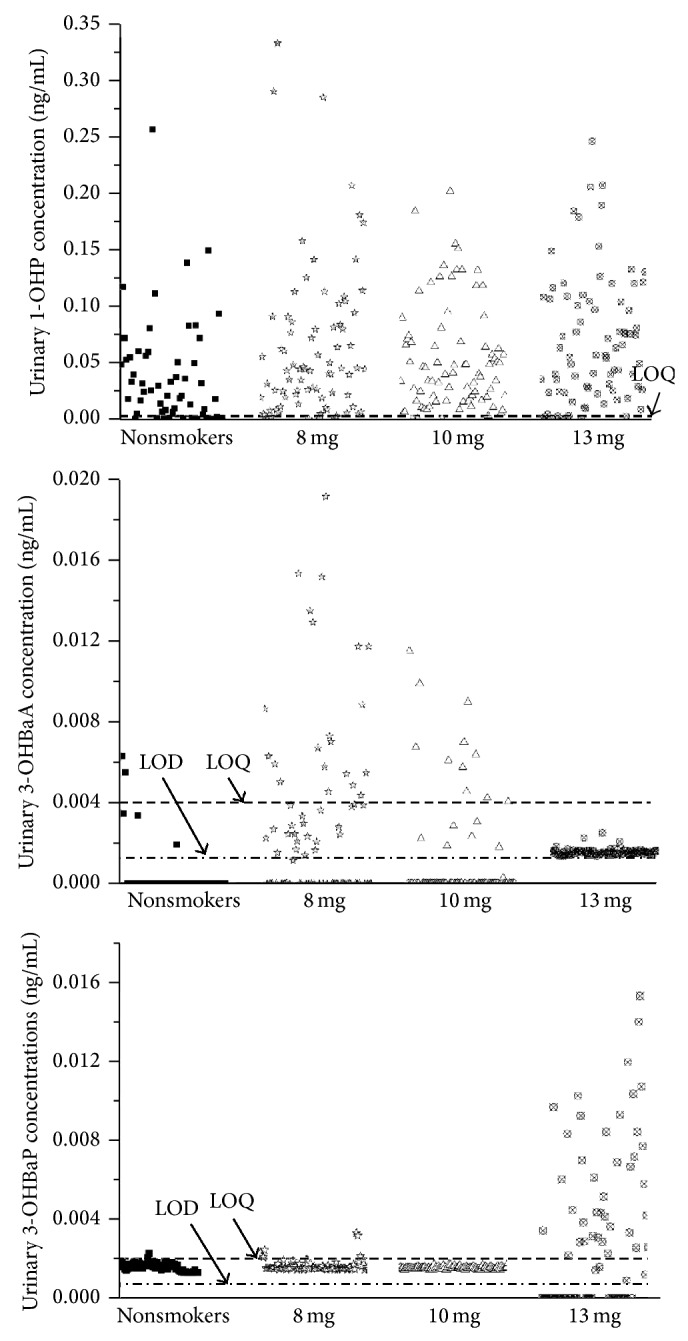
Urinary 1-OHP, 3-OHBaA, and 3-OHBaP concentrations from nonsmokers and smokers.

The proposed method was successfully used to analyze 58 urine samples from 58 nonsmokers and 243 urine samples from 81 smokers (smokers smoked 8 mg, 10 mg, and 13 mg tar cigarettes in three different weeks, and urine samples were then collected every 24 h); the results are shown in Figure 3. After removing the outliers and the values lower than the LOQs from each group, the final results were summarized as geometric means (GMs) and geometric standard deviations (GSDs) (Table 3). With the exceptions of urinary 3-OHBaA in smokers with 13 mg tar cigarettes and 3-OHBaP in smokers with 10 mg tar cigarettes, urinary 1-OHP, 3-OHBaA, and 3-OHBaP levels in smokers were higher than those in nonsmokers.

**Figure 4:**
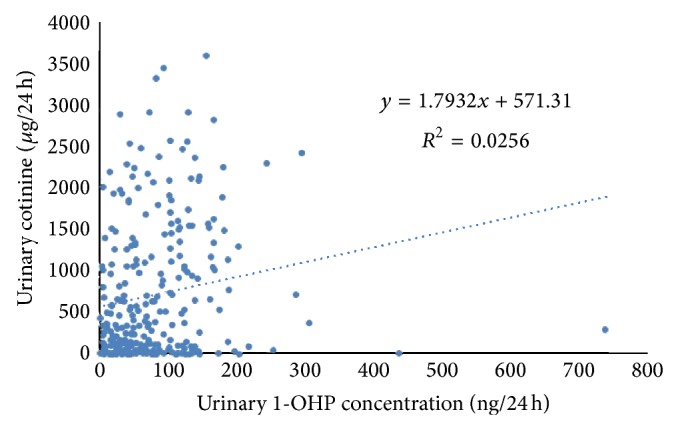
Correlation between urinary cotinine and 1-OHP.

Because spot urine sampling may not provide a valid overview of the entire toxicant exposure profile [38] and is easily influenced by other factors (e.g., diet), urinary 1-OHP, 3-OHBaA, and 3-OHBaP concentrations were adjusted by creatinine excreted in urine at a relatively constant rate through glomerular filtration. The results after the creatinine correction are summarized in Table 4. Urinary 1-OHP and 3-OHBaP levels increased with tar content, while 3-OHBaA levels did not. Significant differences were observed in the urinary 1-OHP levels between smokers with 8 mg tar cigarettes and 10 mg tar cigarettes (*P* < 0.01) and between smokers with 10 mg tar cigarettes and 13 mg tar cigarettes (*P* < 0.05). However, the differences in urinary 3-OHBaA and 3-OHBaP between different groups of smokers were not significant (*P* > 0.05). The correlation between 1-OHP in 24 h urine and the nicotine metabolite cotinine, which is a recognized biomarker for tobacco exposure, was also studied (Figure 4). The correlation between urinary 1-OHP and cotinine was weak (*y* = 1.7932*x* + 571.31, *R* ^2^ = 0.0256).

## 4. Discussion

Pyrene, benzo[a]pyrene, and benz[a]anthracene are ubiquitously present in the environment and cigarette smoke and are associated with a variety of adverse health effects. In mainstream cigarette smoke, the levels of benzo[a]pyrene and benz[a]anthracene ranged from 8.5 to 11.6 ng/cig and 20 to 70 ng/cig [39], respectively. OH-PAHs have been employed as biomarkers for the human exposure assessment of PAHs [32]. In previous studies [30, 31, 40], only the single analyte determination of 1-OHP or 3-OHBaP was carried out. Here, we developed a new LC-APCI-MS/MS method to simultaneously analyze urinary 1-OHP, 3-OHBaP, and 3-OHBaA. The proposed method has good precision and accuracy, very high sensitivity, an extraction procedure suitable for processing large batches of samples, and a relatively short chromatographic run time (12 min).

Urinary 1-OHP concentrations in nonsmokers (58.16 ng/g creatinine) and smokers (66.85 ng/g creatinine) in this study were lower than those reported in France by Lafontaine et al. (84.88 ng/g creatinine and 277.6 ng/g creatinine) [41], which may result from differences in subjects due to their different environments and the type and number of cigarettes used. In Lafontaine's study, blended cigarettes were used, and smokers smoked 15–30 cigarettes per day, which was two times as many as ours. In addition, daily exposure to the sum of benzo[a]pyrene, benz[a]anthracene, chrysene, and benzo[b]fluoranthene was higher in France (estimated at 1.48 ng/kg bw/day in adults and 2.26 ng/kg bw/day in children) [42]. Urinary 3-OHBaA levels in nonsmokers and smokers were lower than those for people in the US over 20 years of age [43]. In the studies of Fan et al. [29] and Lafontaine et al. [41], the average concentrations of 3-OHBaP were 14.23 ng/g creatinine and 33.2 ng/g creatinine, respectively, which were much higher than those observed in this study. This might result from the fact that the subjects in our studies were exposed to extremely low levels of PAHs in the environment.

**Figure 5:**
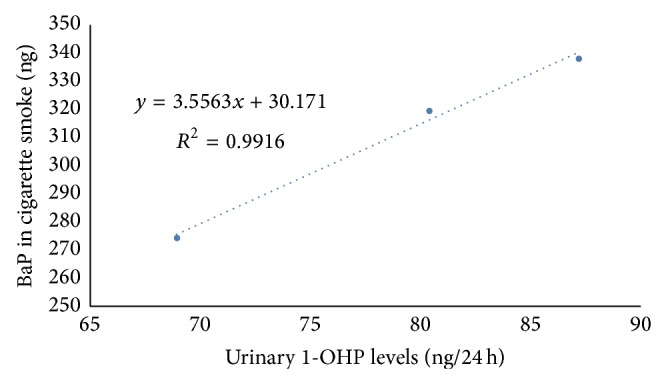
Correlation between urinary 1-OHP and cigarette smoke BaP under Canadian intense machine smoking regime.

After creatinine correction, urinary 1-OHP concentrations in smokers were higher than those in nonsmokers, in agreement with previous reports [41, 44]. In addition, urinary 1-OHP increased with the tar, BaP, or pyrene in mainstream cigarette smoke. Two dose-response models relating 24 h urinary mean 1-OHP concentration and BaP yield in mainstream smoke under the ISO smoking regime and the Canadian intense machine smoking regime were established. The dose-response model between urinary 1-OHP and BaP yield under the ISO smoking regime (93.00, 148.20, and 151.80 ng/15 cigs) was *y* = −0.3322*x* + 157.18 (*R* ^2^ = 0.0086, *n* = 227); under the Canadian intense machine smoking regime (319.05, 274.05, and 337.95 ng/15 cigs), BaP yield was strongly related to urinary 1-OHP (*y* = 3.5563*x* + 30.171, *R* ^2^ = 0.9916, *n* = 227, Figure 5).

## 5. Conclusions

A LC-APCI-MS/MS method for the simultaneous analysis of urinary 1-OHP, 3-OHBaP, and 3-OHBaA was developed and validated. The proposed method has good precision and accuracy, notably high sensitivity, and a relatively short chromatographic run time (12 min). The method was successfully used to assess urinary 1-OHP, 3-OHBaP, and 3-OHBaA in nonsmokers and smokers with different tar yield cigarettes. After creatinine correction, urinary 1-OHP and 3-OHBaP increased with tar and BaP contents in cigarette smoke. Urinary 1-OHP levels were significantly correlated with BaP yield in cigarette smoke under the Canadian intense smoking condition.

## Figures and Tables

**Table 1:** Demographics of subjects.

	Gender	Age (years)	Smoking habits (number)
Nonsmokers (*n* = 58)	Man	22.34 ± 1.46	
Smokers (*n* = 81)	78 men, 3 women	22.95 ± 2.10	9.50 ± 5.30

The results were expressed as mean ± standard deviation.

**Table 2:** Recovery and precision data for 1-OHP, 3-OHBaP, and 3-OHBaA.

	Spiked	Conc.	Recovery	Intraday precision	Interday precision
	ng/mL	ng/mL	*n* = 6, (%)	*n* = 6, (%)	*n* = 6, (%)
1-OHP	0.015	0.016	106.7	6.5	11.4
	0.076	0.080	105.6	5.1	4
	0.23	0.24	104.3	5.5	4

3-OHBaP	0.003	0.002	72.1	8.8	5.9
	0.015	0.012	80.2	4.5	7.3
	0.045	0.036	80.7	5.2	6

3-OHBaA	0.008	0.007	87.5	6.1	8.4
	0.038	0.036	94.7	5.8	6.7
	0.114	0.1135	99.6	3.9	1.8

**Table 3:** Urinary 1-OHP, 3-OHBaP, and 3-OHBaA concentration after removing abnormal value.

Analyte	Nonsmokers	Smokers			
		8 mg	10 mg	13 mg	Total
1-OHP (ng/mL)	0.047 ± 0.048 (*n* = 46)	0.057 ± 0.058 (*n* = 74)	0.047 ± 0.040 (*n* = 79)	0.062 ± 0.048 (*n* = 74)	0.055 ± 0.050 (*n* = 227)
3-OHBaA (ng/mL)	0.006 ± 0.0006 (*n* = 2)	0.008 ± 0.004 (*n* = 23)	0.009 ± 0.009 (*n* = 13)	—	0.008 ± 0.006 (*n* = 36)
3-OHBaP (ng/mL)	0.002 ± 0.0002 (*n* = 2)	0.0024 ± 0.0005 (*n* = 9)	—	0.006 ± 0.003 (*n* = 39)	0.005 ± 0.003 (*n* = 48)

The results were expressed as mean ± standard deviation.

**Table 4:** Urinary 1-OHP, 3-OHBaP, and 3-OHBaA concentration after creatinine correction.

Analyte	Nonsmokers	Smokers			
		8 mg	10 mg	13 mg	Total
1-OHP (ng/g creatinine)	58.16 ± 49.48 (*n* = 46)	62.28 ± 70.58 (*n* = 74)	63.51 ± 68.75 (*n* = 79)	74.88 ± 73.68 (*n* = 74)	66.85 ± 70.91 (*n* = 227)
3-OHBaA (ng/g creatinine)	10.98 ± 4.39 (*n* = 2)	11.01 ± 13.30 (*n* = 23)	9.17 ± 12.89 (*n* = 13)	—	10.35 ± 13.00 (*n* = 36)
3-OHBaP (ng/g creatinine)	1.30 ± 0.20 (*n* = 2)	2.83 ± 1.78 (*n* = 9)	—	6.00 ± 4.44 (*n* = 39)	5.41 ± 4.21 (*n* = 48)

The results were expressed as mean ± standard deviation.
